# Definition of intercultural competence (IC) in undergraduate students at a private university in the USA: A mixed-methods study

**DOI:** 10.1371/journal.pone.0196531

**Published:** 2018-04-26

**Authors:** Lioba Gierke, Nadine Binder, Mark Heckmann, Özen Odağ, Anne Leiser, Karina Karolina Kedzior

**Affiliations:** 1 Institute of Psychology and Transfer, University Bremen, Bremen, Germany; 2 Psychology, Jacobs University, Bremen, Germany; 3 Psychology, Touro College Berlin, Berlin, Germany; Universidade do Porto, Faculdade de Farmácia, PORTUGAL

## Abstract

**Introduction:**

Intercultural competence (IC) is an important skill to be gained from higher education. However, it remains unclear what IC means to students and what factors might influence their definitions of IC. The aim of the current study was to qualitatively assess how students at one higher education institution in the USA define IC and to quantitatively test for relationships among IC components and various demographic characteristics, including intercultural experience and study context. A further aim was to descriptively compare the IC definitions from the US sample with the definitions obtained from another sample of university students in Germany.

**Materials and methods:**

A purposive sample of *n* = 93 undergraduate, second semester students at Dickinson College, USA, participated in the study by completing an online questionnaire. The qualitative data were content-analyzed to define the dimensions of IC. The quantitative data were cluster-analyzed to assess the multivariate relationships among the IC components and the demographic characteristics of the sample.

**Results:**

The most important dimensions of IC were Knowledge, External Outcomes (interaction, communication), and Attitudes (respect, tolerance) according to the US sample. The most frequently chosen dimensions of IC differed between both samples: Knowledge was chosen by the sample in the USA while External Outcomes was chosen by the sample in Germany. Relative to the US sample, significantly more students chose Attitudes, External Outcomes, and Intrapersonal Skills in the sample in Germany. The relationships among IC components and demographic characteristics were only weak in the US sample. A person with IC was rated as Open-minded and Respectful by students who lived predominantly in the USA or Tolerant and Curious by those who lived outside the USA for at least six months.

**Discussion:**

The current results suggest that students residing in two countries (USA or Germany) define IC using similar dimensions. However, IC definitions may depend on the intercultural experience and the current global discourse. Longitudinal studies with representative samples are required to assess how IC definitions change over time.

## Introduction

Internationalization of higher education has become a global phenomenon [1]. One of its central elements is the ‘interest in producing globally competent graduates capable of understanding and functioning in a complex and interconnected world’ ([1], p. 6). Such globally competent graduates are expected to possess intercultural competence (IC) [2]. But what exactly is IC and how can students acquire such a competence? Borrowing from the business expertise on expatriate preparation for international assignments [3], many institutions worldwide offer extracurricular activities, such as training in IC (for example see [4]) designed to translate the internationalization policies into practice [1]. However, such training programs as well as institutional mission statements and policies often do not explicitly define IC nor explain how it can be acquired [5–7]. Therefore, the meaning of IC needs to be clarified before the internationalization policies can be developed and successfully implemented in higher education [8]. This is particularly important because the global mobility is likely to increase in the future and the virtual mobility resulting from the new technologies already contributes to intercultural experiences ‘at home’ [7].

The concept of IC has been defined predominantly on the theoretical level in the western educational contexts and in the academic discourse in the non-western cultures (for review see [9, 10]). The theoretical models refer to IC using its communicative, cognitive or global aspects, such as *intercultural communicative competence*, *cultural intelligence*, or *global citizenship*, among others [8]. The multidimensionality of IC is also evident in terms of its association with various attitudes, knowledge, skills, and outcomes and its dependence on the context and identity in the academic discourse [10].

Reasonably little effort has been made to define IC empirically meaning that the validity and generalizability of the theoretical models is largely unknown so far [9]. A widely accepted definition empirically derived from the opinions of scholars and administrators in the USA states that IC is ‘one’s ability to communicate effectively and appropriately in intercultural situations based on one’s intercultural knowledge, skills, and attitudes’ ([2], p. 248). The categories embedded in this definition were visualized in the Pyramid Model of Intercultural Competence to emphasize that IC is a developmental process [2].

Although the Pyramid Model [2] captures the multidimensionality of IC according to the providers of higher education (the experts: scholars/administrators), it may not necessarily reflect the opinions of the receivers of higher education (the students). In fact, while the experts in the USA focused on the understanding of *own* culture in the Pyramid Model study [2], the university students (domestic and international) in Australia, Hong Kong, and Germany noted that the understanding of *other* cultures is an important requirement for IC [11–13]. There could be a number of reasons for this difference in focus of IC definitions between the experts and the students. First, an obvious candidate is own cultural background that was reasonably homogeneous among the experts (predominantly US-based) in the Pyramid Model study [2] and highly heterogeneous among the students [11–13]. Second, IC appears to be strongly context dependent according to data from undergraduate students studying at two universities in the same city in Germany (a private, international university [13] or a public university [14]). Specifically, the international students defined IC in terms of the external outcomes with practical connotations (interaction and communication) required for living and studying on the international university campus [13]. In contrast, the domestic students with less international contacts focused on ‘the correct’ (socially-acceptable) attitudes (tolerance, acceptance, openness) as the main components of IC [14]. Third, other demographic factors, personality traits, and intercultural experience at home or abroad could affect student definitions of IC. In general, students exposed to intercultural issues in the home-based classroom or those participating in activities abroad report initial difficulties followed by enormous development and change depending on various demographic and personality traits (see [15]). Fourth, researching of IC is a methodologically difficult task from the conceptual point of view [16]. A comprehensive theory of IC addressing its components, structure, and validity in real encounters is still missing [16]. Possibly for this reason the majority of studies so far explored the meaning of IC using qualitative approaches. Although a number of quantitative assessment tools exist, they are designed to measure specific outcomes of IC, such as cultural knowledge or language proficiency [17]. Utilizing of mixed methods approaches might be particularly useful to study IC. Specifically, the qualitative data could be used to describe the components and structure of IC while the quantitative data could be used to investigate the relationships among IC components and other factors. Overall, additional research is required to study how university students define IC and what factors might influence their definitions of IC.

The current study seeks to address these issues by gathering mixed (qualitative and quantitative) data regarding the definition of IC from a sample of undergraduate students who, like the international student sample in Germany [13], live and study on a small, private university campus in the USA (Dickinson College, Pennsylvania). It is interesting to examine how such students define IC because the USA has a long tradition of exploring the role of migration and cultural diversity in higher education and is one of the most popular study-abroad destinations in the world. Furthermore, the Pyramid Model [2] was also developed in the USA.

The current study has three specific aims: (1) to obtain a qualitative definition of IC in a sample of undergraduate university students in the USA using the same methods as in the study with the international students in Germany [13], (2) to descriptively compare the definitions of IC in both student samples; and (3) to quantitatively investigate the relationships among IC components and demographic factors.

## Materials and methods

### Participants

The participants were recruited via email and a word of mouth. The study used a purposive sampling strategy similar to that utilized in the study in Germany [13] to allow for a comparison between both studies. All participants had to meet the following criteria: 1. undergraduate student, 2. second semester student, 3. student enrolled at Dickinson College, USA. Following a written informed consent (see Document A in the S1 File of the Supporting Information), 101 participants (14% of 706 students in their second semester of studies) participated in the study in spring 2016. Since all students were in their freshman year at college they had not yet chosen a study major at the time of data collection.

### Questionnaire

The study involved a completion of two anonymous questionnaires in English administered online using Qualtrics software. Questionnaire 1 (Document B in the S1 File of the Supporting Information) was adopted from the study in Germany [13] and included a single open-ended question requiring the students to define IC in their own words and eight demographic questions regarding nationality and intercultural experience. Eleven participants were excluded due to missing data (failing to answer at least one question). The final sample of *n* = 93 participants provided complete responses to all questions.

Questionnaire 2 (see Document C in the S1 File of the Supporting Information) was self-developed for the purposes of this study. The questionnaire consisted of semantic differential items requiring the students to quantitatively describe a person possessing IC. The scale consisted of 21 bipolar adjective pairs, such as ‘tolerant—intolerant’, arranged in two columns and separated by a scale from 1 to 6 without neutral option to prevent satisficing (scores of 1 and 6 meaning ‘extremely’, 2 and 5 meaning ‘moderately’, 3 and 4 meaning ‘slightly’). The adjectives were derived from our previous qualitative definitions of IC [13]. The positive and negative adjectives were randomly distributed in both columns to minimize the response bias. The participants were asked to consider an interculturally-competent person and place a cross on a rating (from 1 to 6) that best describes such a person (for example, to what extend such a person is tolerant or intolerant). Once data were collected the responses were recoded such that 1 indicated extremely negative attributes (for example, extremely intolerant) and 6 indicated extremely positive attributes (for example, extremely tolerant). In addition, participants were asked to select three most important adjectives (from the 21 adjective pairs) that best describe an interculturally-competent person. Self-ratings of own IC were not investigated since we expected that such ratings would have been inflated in desired directions due to the social desirability bias.

### Procedure

Following the approval of the Ethics Committee at Dickinson College (IRB ID 444, approved on March 1, 2016), the questionnaires were pilot-tested with five students to ensure that they comprehended the questions prior to the data collection. Once this was assured, all participants electronically signed an informed consent form and completed both questionnaires online. The mixed-methods approach was applied sequentially to prevent any carry-over effects. Specifically, participants were asked to complete the qualitative questionnaire 1 first (to define IC in their own words) followed by the quantitative questionnaire 2 (to rate an interculturally-competent person using a list of adjectives). There was no option to go back and modify own responses on questionnaire 1 after it was completed and the participant started on questionnaire 2. Data collection took part in a psychology laboratory at Dickinson College during business hours in March 2016. Participants were debriefed (see Document D in the S1 File of the Supporting Information) and received course credits for participation in the study.

## Results

### Participant characteristics

#### Sample in the USA

Most of the 93 participants were young (about 19 years old), female, undergraduate university students in their second semester at Dickinson College, USA (Table 1). The majority of the sample had a US nationality and attended local public (not international) high schools with English as the language of instruction. Although 61% reported having taken part in any IC-related workshops or courses, only a minority (25%) had ever lived outside the USA for more than six months and only 23% had a study abroad experience (Table 1).

**Table 1:** Demographic characteristics of students in the US sample.

Demographic characteristics		Sample size *n* (% of 93)
Age (mean ± 1 standard deviation); range		19±1; 18–20
Gender	Male	23 (25%)
	Female	70 (75%)
High school attended	Local school	81 (87%)
	International school	12 (13%)
Language at high school	English	85 (91%)
	Other	8 (9%)
Nationality	USA	71 (76%)
	Other	22 (24%)
Lived in countries other than the USA for at least 6 months	Yes	23 (25%)
	No	70 (75%)
Study Abroad experience	Yes	21 (23%)
	No	72 (77%)
Took part in intercultural competence workshop/course	Yes	57 (61%)
	No	36 (39%)

#### Sample in Germany

This sample was already described in our previous study [13]. The sample of 130 participants in Germany was similar to the US sample in terms of the following demographic characteristics: age (on average 19 years old), gender (majority- 62%- female), high school type (majority- 71%- at local public schools), and university studies and structure (undergraduate, 2^nd^ semester students at a small, campus-based, international university- Jacobs University Bremen, Germany) [13]. Unlike the homogenous (mostly US) nationality in the current sample, the sample in Germany was international with majority (58%) reporting a non-German nationality [13]. Furthermore, more participants in the study in Germany attended IC-related workshops (100% vs. 61% in the US sample) and had study abroad experience prior to university (42% vs. 23% in the US sample) [13].

### Qualitative IC definition according to students in the US sample

The qualitative data were analyzed using the content analysis according to the guidelines by Schreier [18]. The content analysis was done using a coding frame from our previous study [13]. The coding frame was deductively derived from the Pyramid Model [2] and consisted of six main dimensions (Attitudes, Knowledge, Inter- and Intrapersonal Skills, Internal and External Outcomes) and multiple subcategories of each dimension (for example, ‘Respect’ and ‘Tolerance’ as subcategories of Attitudes). An additional subcategory of Attitudes, ‘Equality of Cultures’, was inductively derived from the current data.

The responses to the open-ended definitions of IC were segmented into coding units (such as one concept or one sentence if a participant provided a multi-sentence definition). Each coding unit was then coded by matching it with a single subcategory from the coding frame. All data were coded independently by two authors (LG and AL) to assure a high inter-rater agreement. Any inconsistencies in terms of assigning different codes to the same coding units were resolved by consensus during a consultation with a third author (NB), who has advanced experience in coding of IC data, until 100% agreement was reached. The final data were summarized in terms of frequencies with which the participants reported the main dimensions and their subcategories in the IC definitions.

Nearly half of the US sample defined IC predominantly in terms of Knowledge, with the main focus on intercultural awareness (Table 2). The other two frequently mentioned dimensions of IC were External Outcomes (especially effective/appropriate interaction) and Attitudes (particularly respect and tolerance/acceptance; Table 2). The least important dimensions of IC were Internal Outcomes, and Intra- and Interpersonal Skills (Table 2).

**Table 2:** Dimensions of IC according to students in the US sample.

Dimensions (*n*; % of 93 participants)	Subcategories (*n*; % of coding units for each dimension)
1. Knowledge (*n* = 47; 51%)	Intercultural Awareness (*n* = 41; 69%) Understanding Other’s World Views (*n* = 11; 18%) Culture-Specific Knowledge (*n* = 4; 7%) Culture Self Identity/Awareness (*n* = 2; 3%) Understanding Other’s Behaviors (*n* = 2; 3%)
2. External Outcomes (*n* = 26; 28%)	Effective/Appropriate Interaction (*n* = 11; 38%) Effective/Appropriate Communication (*n* = 7; 24%) Integration (*n* = 5; 17%) Collaboration/Cooperation (*n* = 3; 10%) Offence Prevention (*n* = 2; 7%) Non-Discrimination (*n* = 1; 4%)
3. Attitudes (*n* = 22; 24%)	Respect (*n* = 11; 38%) Tolerance/Acceptance (*n* = 9; 31%) Openness (*n* = 4; 14%) Curiosity/Discovery (*n* = 3; 10%) Equality of Cultures (*n* = 2; 7%)
4. Internal Outcomes (*n* = 12; 13%)	Informed Frame of Reference (*n* = 5; 36%) General Adaptability/Adjustment (*n* = 4; 29%) Empathy (*n* = 2; 14%) Internal Outcomes Miscellaneous (*n* = 2; 14%) Ethnorelativism (*n* = 1; 7%)
5. Intrapersonal Skills (*n* = 5; 5%)	Culture Detection (*n* = 2; 33%) Judgment Inhibition (*n* = 2; 33%) Critical Thinking Skills (*n* = 1; 17%) Intrapersonal Skills Miscellaneous (*n* = 1; 17%)
6. Interpersonal Skills (*n* = 2; 2%)	Interpersonal Skills Miscellaneous (*n* = 2; 100%)

Note. The scores exceed 100% because most participants provided IC definitions consisting of multiple dimensions and/or subcategories.

### Descriptive comparison of IC definitions according to samples in the USA vs. Germany

We compared the dimensions of IC definitions between the samples in the current study and the study in Germany [13] using descriptive statistics (frequency of responses) and the univariate chi-square tests (see Tables A and B in the S1 File of the Supporting Information). Relative to the US sample, the sample of students in Germany [13] was significantly more international (in terms of the non-German nationality) and had significantly more study abroad experience (Table B in the S1 File of the Supporting Information).

**Fig 1:**
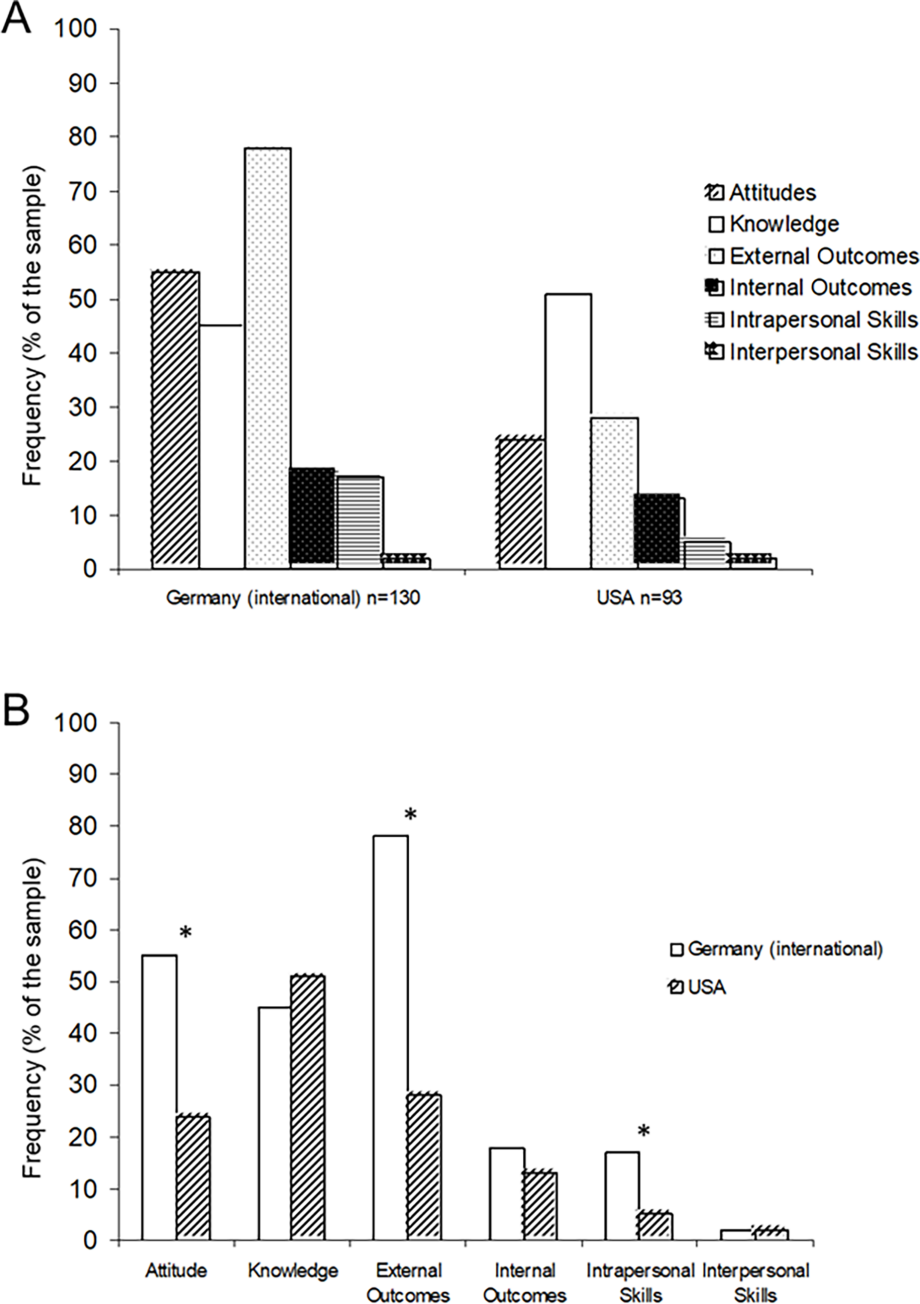
Dimensions of IC in two samples of undergraduate university students. Note. A. IC dimensions within each sample: the sample in Germany [13] and the current sample in the USA; B. IC dimensions between both samples. *p < .05.

The international students in Germany [13] and the students in the USA defined IC predominantly in terms of Attitudes, Knowledge, and External Outcomes dimensions (Fig 1A; Table A in the S1 File of the Supporting Information). However, the frequency of responses showed that each of the samples tended to focus on a different dimension of IC: the sample in Germany [13] chose predominantly External Outcomes while the sample in the USA chose predominantly Knowledge (Fig 1A). A comparison between both studies revealed that IC was defined in terms of Attitudes, External Outcomes, and Intrapersonal Skills significantly more often by the sample in Germany [13] than the sample in the USA (Fig 1B; Table B in the S1 File of the Supporting Information). There were no differences between the samples in terms of Knowledge, Internal Outcomes, and Interpersonal Skills (Fig 1B; Table B in the S1 File of the Supporting Information).

### Quantitative relationships among IC components and demographic factors in the US sample

**Fig 2:**
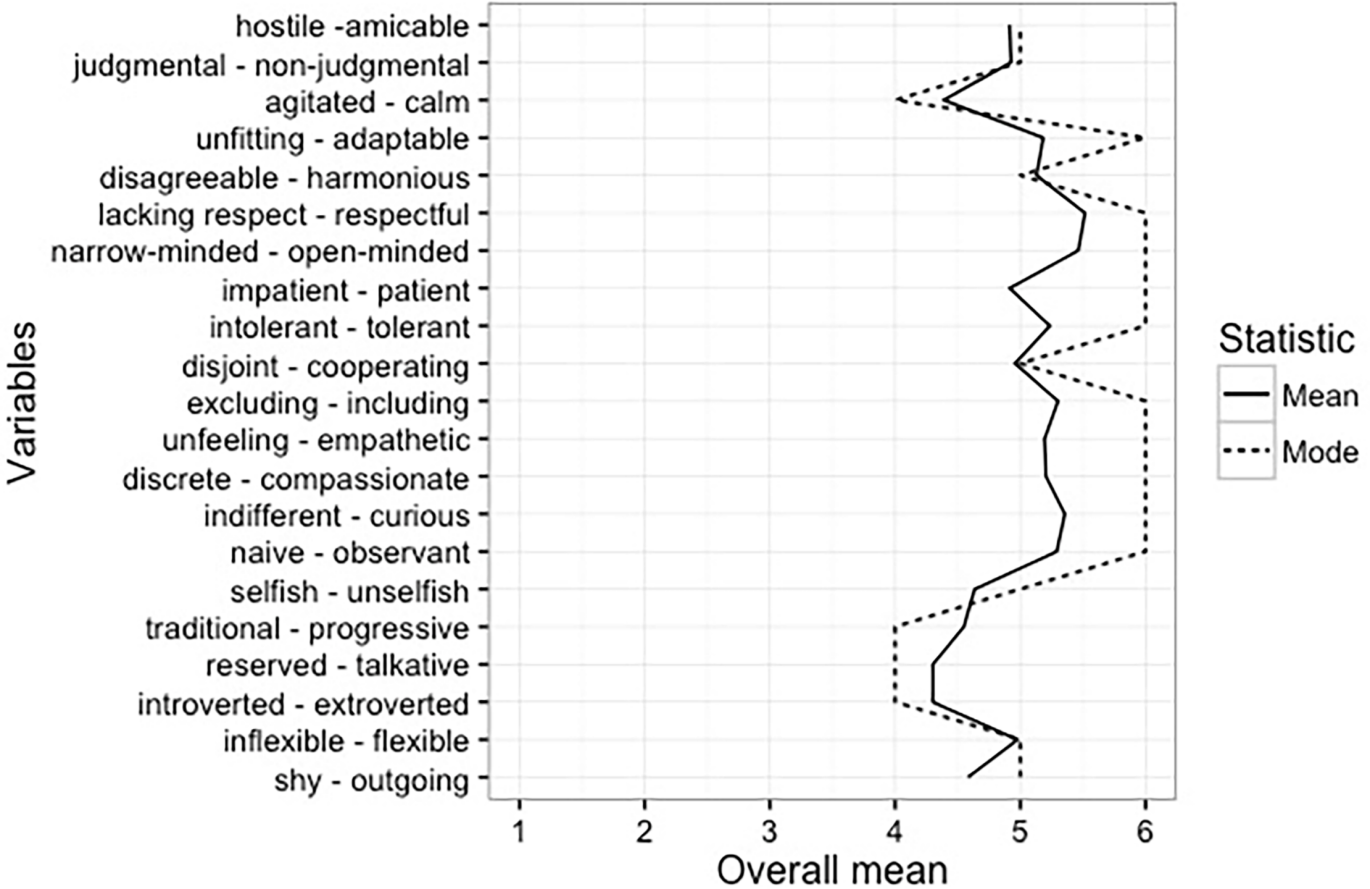
Rating of adjectives describing an interculturally-competent person according to the US sample (*n* = 93). Note. There were two modes for the item judgmental vs. non-judgmental (5 and 6; only 5 is shown on the figure).

The quantitative responses on the semantic differential scale were analyzed using R 3.3.1 and SPSS-22. According to the mode of responses, the majority of students in the US sample described an interculturally-competent person as (strongly) non-judgmental, adaptable, respectful, open-minded, patient, tolerant, including, empathetic, compassionate, curious, and observant (Fig 2).

The three most important characteristics of an interculturally-competent person from Fig 2 are: Open-Minded (listed by 64% of the sample), Respectful (34% of the sample), and Observant/Tolerant/Curious (23–27% of the sample; also see Supporting Information). All these characteristics correspond to the Attitudes dimension of the coding frame from the content analysis (Table 2).

Cluster analysis with non-negative matrix factorization (NMF) was used to identify groups of participants who chose similar adjectives to describe an interculturally-competent person. NMF is a dimensionality reduction technique that attempts to find latent patterns in data [19]. It can be used for clustering by identifying groups of cases which are associated with (or highly load on) one of the latent features (clusters) [20]. Due to incomplete data, two participants were removed from the cluster analysis.

A four-cluster solution was subjectively chosen as the best solution because each cluster was associated with one dominant loading on a different adjective describing an interculturally-competent person (Table C and Figure A in the S1 File of the Supporting Information). Comparing the demographic characteristics of participants in each cluster suggests that only relatively weak trends exist in the current data (Table 3). Specific adjectives describing an interculturally-competent person were chosen by participants with the following demographic characteristics:

- Open-Minded was chosen predominantly by those with US nationality who lived mostly in the USA
- Respectful was chosen predominantly by females with US nationality, who lived mostly in the USA
- Tolerant was chosen predominantly by those who lived outside the USA for at least six months, had study abroad experience and participated in IC workshops/courses
- Curious was chosen predominantly by males who lived outside the USA for at least six months and participated in IC workshops/courses.

There was no clear pattern in demographic characteristics of participants who chose the adjective Observant.

**Table 3:** Demographic characteristics of students in the US sample who described an interculturally-competent person as tolerant, curious, open-minded, and respectful.

Demographic characteristic	Adjective with the highest loading			
	Cluster 1	Cluster 2	Cluster 3	Cluster 4
	Tolerant	Curious	Open-minded	Respectful
	% of *n* = 23	% of *n* = 17	% of *n* = 26	% of *n* = 25
US nationality	70	77	81	76
Lived outside the USA	35	29	19	20
Study abroad	48	12	15	16
IC workshop	74	65	50	60
Male	26	35	27	16
Age	18	18	18	18

## Discussion

The current study adds the empirical evidence regarding IC from the perspective of students in higher education. Although a universal definition of IC may not exist, it seems that the samples of students in the USA, the international students in Germany, and the students in other empirical studies (reviewed below) place a special focus on the role of Knowledge and External Outcomes (interaction, communication) when defining IC. Student definitions of IC may depend on their intercultural experience although only weak relationships were found among the IC components and the demographic factors in the US sample.

### What is IC according to university students?

University students in the current US sample and in the international sample in Germany [13] define IC using dimensions included in the Pyramid Model [2], confirming the validity of the model. Both student samples especially focus on Knowledge (Intercultural Awareness and Understanding of Others’ View Points), External Outcomes (Interaction, Communication), and Attitudes (Tolerance/Acceptance, Respect).

The Knowledge dimension (including Intercultural Awareness and Understanding Others’ World Views) was probably chosen because the students in the current study and in the study in Germany [13] participated in IC-related activities, such as workshops, study courses or training. The content of such activities might have emphasized the importance of cultural knowledge for development of IC. Indeed, internationalization of curriculum by offering IC-related content already at home allows students to gain new knowledge and to critically reflect about global issues (see [15]). The focus on Knowledge is also in line with the opinions of the mostly US-based experts in the Pyramid Model study [2]. As already argued elsewhere [13], the focus on cultural knowledge could also result from the widespread use of online social networks among students, particularly in the USA, where 78% of the population had a social network profile in the year of data collection, 2016 [21]. Social networks, such as ‘Facebook’, easily promote intercultural communication and thus allow opportunities to gain cultural knowledge already at home [22, 23]. Future studies should systematically examine the influence of online social networks on IC development.

Focus on the Knowledge and the External Outcomes (Interaction/Communication) dimensions could also mean that students associate IC with the linguistic skills. Indeed, all students in the current study were required to study a foreign language for at least three semesters during their undergraduate degree at Dickinson College while the majority of students in the study in Germany [13] spoke at least two languages. Other studies report that students focus on linguistic skills when defining IC [11] or when critically reflecting about own IC development following international exchanges [24]. Bilingualism is also positively associated with intercultural communication competence (ICC) that is considered a core component of IC [25]. Among other benefits, studying a foreign language helps to identify with the target culture through increased communication and culture-specific knowledge [26]. In addition, cognitive strategies learned in language classes, including identification, abstraction and comparison, could be useful tools in developing cognitive skills related to IC. These skills may broaden student perceptions and lead to higher levels of abstraction, which are needed for effective intercultural communication when facing new situations [27]. The impact of bi- or multilingualism on IC in higher education should be explored in further research.

Choosing Knowledge, External Outcomes, and Attitudes could also be related to the international experience or exposure of the students. About a quarter of the current US-sample reported a foreign nationality, having lived in another country than the USA for at least six months or having studied abroad prior to enrolling at the university while the sample in Germany included predominantly non-German students who came to study at an international university in Germany [13]. On the one hand, students and educators alike report that international experience, including studying, placements, internships, or volunteering abroad, can be life changing and contributes to enormous personal gains that foster IC development (for specific examples see [15]). Studying abroad is associated with development of cognitive, affective, and behavioral skills, including non-verbal communication, basic living skills, and critical understanding of values and attitudes [28] as well as increases in ICC [29] and world-mindedness [30]. World-mindedness could be equivalent to the subcategory of Knowledge (Understanding Others’ World Views) frequently mentioned by students in both samples because ‘worldminded individuals are more likely to see viewpoints that differ from their own ethnic, national, or religious perspectives as valuable’ ([30], p. 58). The intercultural experience arising from living in different cultures can lead to a growth in a ‘global citizenship’ [31]. Such citizenship includes being aware of global events and valuing of diversity (corresponding to our subcategories of Knowledge: Intercultural Awareness and Understanding Others’ World Views and Behaviors) as well as an improvement in ICC (being part of External Outcomes dimension) [31]. On the other hand, the concept of the ‘global citizenship’ and the value of study abroad have been challenged by a number of authors (see [15, 32]). Overall, direct observations of classroom instructors (see [15]) and empirical assessment of students suggest that the mere exposure, such as study abroad or experience of international study environment at home are insufficient for development and maturation of IC [28, 32, 33]. In fact, well-designed pedagogical opportunities at home may contribute more to the critical reflection regarding culture and IC development than attending classes with international students or participating in study abroad programs [34, 35]. Domestic students emphasize that the knowledge of languages, cultural norms, and pedagogical systems is required for successful interactions between domestic and international students at home [36]. For example, domestic students in the UK are afraid of or avoid contacts with international students due to language barriers, the high perceived risk of causing offence or using politically incorrect language, and the risk to their academic performance related to different academic backgrounds and work-orientation in group-work situations [36]. Interestingly, all these issues mentioned by the UK students are indeed reflected in the IC definitions of our student sample in the USA and the international sample in Germany [13] in terms of the Knowledge, External Outcomes, and Attitudes dimensions. Therefore, these components of IC may need to be addressed in mission statements and internationalization policies as requirements for successful IC development in higher education.

### Are the differences in IC definitions between two student samples meaningful?

Although students in the US sample and the international students in Germany [13] mentioned the same dimensions of IC, the importance of each dimension was different in both samples according to their frequency of responses. The US sample defined IC mostly in terms of Knowledge (51%), External Outcomes (28%), and Attitudes (24%). In contrast, the international students in Germany [13] focused on External Outcomes (78%), Attitudes (55%), and Knowledge (45%). Furthermore, relative to the US sample, significantly more students in the study in Germany [13] noted that Attitudes, External Outcomes, and Intrapersonal Skills are important dimensions of IC. It is unclear if these differences are meaningful since they were explored mostly descriptively or with simple univariate chi-square tests. They are clearly surprising (even on a descriptive level) because both samples lived and studied on small, residential university campuses at the time of data collection. Thus, their context of living and studying was similar. If meaningful, these differences could be attributable to at least two issues. First, the intercultural experience/exposure of students might have resulted in different levels of IC development in both samples. A stronger emphasis on Knowledge (located on the middle tier of the Pyramid Model [2]) rather than the other dimensions suggests that students in the US sample are on the intermediate level of IC development. Although living in a culturally-diverse country (the USA) the sample was reasonably homogeneous in terms of their nationality (mostly US-American) and low international experience (predominantly lived in the USA only and had little study abroad experience). In contrast, the international students in Germany [13] reported highly heterogeneous nationalities and were exposed to other cultures on daily bases while living and studying on a small university campus. Thus, the international sample could be considered more ‘culturally-advanced’ because they defined IC predominantly in terms of External Outcomes that are located on the top tier of the Pyramid Model [2]. Furthermore, the sample of students in Germany [13] probably requires External Outcomes and appropriate Attitudes to successfully integrate and study on a small but highly intercultural university campus relative to the significantly less international sample of students in the US. Second, IC definition may be affected by the current events in the media. This assumption is supported by the additional inductive subcategory in the coding scheme–*equality of cultures–*required to capture specific elements of IC definitions in the US sample relative to the coding scheme used in the study in Germany [13]. The new category probably emerged due to the timing of data collection during the hot debate regarding race and migration in the US media surrounding the 2016 presidential elections. In contrast, the data in the study in Germany [13] were collected shortly before the issues of migration or the refugee crisis in Europe have entered the extensive media discourse in Germany. Therefore, the IC definitions could have been confounded by history and current culture-dependent events (such as political discussions regarding race or migration in the media) rather than meaningful differences between the two samples. Longitudinal data are required to test how IC develops over time and if and how IC definitions are influenced by different local and global events at the time of data collection. The implication of the potential changes in the meaning of IC is that policies and mission statements in higher education may also need to be regularly revised and updated.

### Quantitative approach to defining IC in the US sample

Our mixed-methods approach revealed that students noted different dimensions of IC (predominantly Knowledge) in their qualitative responses relative to their quantitative ratings to describe attributes of a person possessing IC (predominantly emphasizing appropriate Attitudes). As explained above, these results suggest that students in the current sample were probably still developing their own IC. Therefore, they have focused on the more fundamental requirements (Knowledge and Attitudes from the lower tiers of the Pyramid Model [2]) to describe IC in general and in another person. These results also suggest that understanding of IC might differ depending on a) the target to be defined/evaluated and b) the method(s) of IC assessment. First, it is plausible to assume that IC definitions are affected by the extent to which they are person-specific. Describing an imagined person might lead to different IC ratings than describing IC more generally. Second, the differences we observed by using two methodological approaches are also indicative of the power of mixed methods approaches in capturing a more elaborate picture. According to this understanding of mixed methods, qualitative and quantitative methods of data collection are likely to produce different accounts, as they constitute different windows to external reality. The differences, then, do not represent contradictions, but complement each other toward a more comprehensive understanding of the phenomenon under scrutiny [37]. Future studies could be further extended by incorporating qualitative interviews to corroborate on written qualitative statements and quantitative ratings.

Furthermore, there were only weak trends in the quantitative data suggesting that students tended to focus on different aspects of Attitudes when describing an interculturally-competent person, perhaps depending on own intercultural experience. Specifically, the most important characteristics of an interculturally-competent person were Open-Minded and Respectful according to students with presumably less intercultural experience (those who lived predominantly in the USA) and Tolerant and Curious according to students with more intercultural experience (those who lived outside the USA for at least six months, had study abroad experience, and participated in IC-related courses or workshops). These trends imply that individuals with less intercultural experience have a theoretical understanding of what IC entails, including being open and respectful toward people from other cultures. Individuals with more intercultural experience, by contrast, appear to be more familiar with what it takes to actually interact with someone from a different culture, including tolerance to behaviors that are unexpected, different, and potentially daunting. Individuals with more intercultural experience, therefore, define IC as being able to tolerate these ambiguities and still remain curious. Although interesting, the trends in the data were weak possibly due to the difficulties in quantifying the intercultural experience. Students in the current globalized world do not necessarily need to leave their own culture to come in close contact with other cultures. A review of research on IC over the course of ten years revealed a change in the definition of culture from the national level in the past to the global level in the current times [38]. Such a broader concept of global culture allows the individuals to experience different cultures they affiliate with even within one country [39] and to develop multicultural personalities [40]. More research is required to quantify the extend of intercultural experience and the relationships among such experience and IC components.

Three attributes of interculturally-competent person, including Open-mindedness, Tolerance, and Curiosity may represent highly relevant aspects of IC. Other research has shown that people who have lived abroad report higher open-mindedness [40]. Open-mindedness may in fact constitute an important outcome of living-abroad as well as an important facet of IC [40]. In a similar vein, the emphasis on Tolerance and Curiosity in the students’ quantitative accounts of IC might represent important outcomes of IC-related courses and workshops or of own intercultural experience. Indeed, a curriculum that includes cross-cultural content has been shown to increase cultural sensitivity in students [41]. Furthermore, not only intercultural experience but rather own personality might account for the trends observed in the current data (see [15]). In fact, the best predictor of IC after study abroad was a pre-college IC score [32] suggesting that the benefits from exposure to other cultures probably depend on factors, such as personality traits and other demographic characteristics (see [15]). For example, one of the Big Five personality characteristics, openness to new experience, has been shown to be essential for effective functioning in diverse cultural settings [42] and positively correlates with cultural empathy [33]. In sum, our quantitative data imply that important relationships may exist among IC components, intercultural experience as well as other characteristics, such as demographics and personality traits. These relationships would need to be tested using larger and more representative samples of students.

## Limitations

The current study has several limitations. First, similar to the previous studies in Germany [13, 14], the current sample was drawn from only one higher education institution in the USA using a purposive sampling strategy. Therefore, the results from this small sample cannot be considered representative. Instead, the current study provides further empirical evidence required to understand how students in higher education define IC. Second, a qualitative data collection method using short, written responses to a single open-ended question might have been insufficient for students to adequately elaborate on their understanding of IC. Semi-structured interviews might have been more effective in providing a holistic picture of IC and should be considered in future research [43]. Third, we critically question whether the quantitative ratings of IC used in our scale best reflect the complexity of the IC construct. Although not ideal, the strength of our quantitative approach is that it allowed us to cluster IC components with demographic characteristics of students. While the data patterns in our sample were only weak, more meaningful differences among clusters might emerge in larger, representative samples. Fourth, the data collected in our study were cross-sectional. Longitudinal (pre-post) designs could help to detect maturation and development of IC and to establish causality in the future studies [16]. Finally, we compared the IC dimensions between the samples in the current study and the study in Germany [13] mostly descriptively using the frequency of responses and the univariate chi-square tests. These simplistic methods were chosen because different demographic characteristics were collected in both studies and the data in both samples were collected at two different points in time. More complex methods of data analysis, such as propensity score matching, could be used to investigate what covariates affect the IC definitions providing that the same covariates are collected and that the studies with different samples are conducted at the same time to eliminate the effects of latent variables, such as history or current global events.

## Conclusion

Our results show that university students define IC in terms of Knowledge, External Outcomes, and Attitudes irrespective of their nationality (international or predominantly US-American) and country of current residence (USA or Germany). The descriptive differences in foci of IC definitions between student samples in two countries (Knowledge in the USA and External Outcomes in Germany) may be related to either the intercultural experience or be influenced by global issues at the time of data collection. Longitudinal studies with large, representative samples are required to assess how IC develops and how its definition changes over time. Furthermore, university students focus on different dimensions of IC when defining the concept qualitatively (focusing on Knowledge) than when asked to quantitatively rate IC in another person (focusing on Attitudes). Understanding of the unique context, cultural background, and intercultural experience might be crucial for measures to support IC development in university students in terms of context-specific learning objectives for training, workshops as well as university-wide policies and mission statements. The quantitative ratings provide some support for the effect of such workshops and study abroad experience on IC definitions.

Taken together, these results suggest that IC definitions are not monolithic and fixed. Instead, IC definitions are dependent on a number of demographic and experiential features of the individual and highly context-specific. If a uniform IC definition does not exist then the higher educational institutions need to be explicit in what aspects of IC their students are required to gain. Furthermore, internationalization policies and intercultural training in the context of higher education need to be carefully designed to meet the needs of student groups with different cultural backgrounds and levels of intercultural experience.

## Supporting information

S1 File This file includes 4 documents (A: Consent form; B: Questionnaire 1; C: Questionnaire 2; D: Debriefing form), 3 tables (A: Dimensions of IC in two samples of undergraduate university students; B: Comparison in dimensions of IC between two samples of undergraduate university students; C: Ranks of the most important characteristics of an interculturally-competent person), and 1 figure (A: Heatmap of a four-cluster solution).
(DOCX)

S2 File A zip file including the data files from the current study. (ZIP)
