# Supplementary material for: Definition of intercultural competence (IC) in undergraduate students at a private university in the USA: A mixed-methods study
Source: PLoS One. 2018 Apr 26;13(4):e0196531. doi: 10.1371/journal.pone.0196531 (PMC5919648; doi:10.1371/journal.pone.0196531)
Supplement: S1 File — This file includes 4 documents (A: Consent form; B: Questionnaire 1; C: Questionnaire 2; D: Debriefing form), 3 tables (A: Dimensions of IC in two samples of undergraduate university students; B: Comparison in dimensions of IC between two samples of undergraduate university students; C: Ranks of the most important characteristics of an interculturally-competent person), and 1 figure (A: Heatmap of a four-cluster solution). (DOCX) [file pone.0196531.s001.docx]

**Supporting Information File S1**

Gierke L, Binder N, Heckmann M, Odag Ö, Leiser A, Kedzior KK. Definition of intercultural competence (IC) in undergraduate students at a private university in the USA: a mixed-methods study. Plos One. 2018. doi: 10.1371/journal.pone.0196531.

## Document A. Consent form

**CONSENT TO PARTICIPATE IN A RESEARCH STUDY**

Below is a description of the research procedures and an explanation of your rights as a research participant.  You should read this information carefully. If you agree to participate, you will sign in the space provided to indicate that you have read and understand the information on this consent form. You are entitled to and will receive a copy of this form.

You have been asked to participate in a research study conducted by Lioba Gierke ([lioba.gierke@web.de](mailto:lioba.gierke@web.de)), a student in the Department of Psychology at the University of Bremen. The supervisor for this study is Professor Karina Karolina De Santis ([desantis@uni-bremen.de](mailto:desantis@uni-bremen.de)) from the University of Bremen.

**WHAT THE STUDY IS ABOUT:**
The purpose of this research study is to investigate intercultural competence and the understanding of such.

**WHAT WE WILL ASK YOU TO DO:**
During this study, you will receive one questionnaire, asking about your demographic characteristics and an open-end question, requiring a definition of intercultural competence in your own words. In the second step, you will receive a questionnaire with a list of adjectives, asking you to decide and mark to what extend each adjective describes an interculturally-competent person.

**DURATION AND LOCATION OF THE STUDY:**
Your participation in this study will involve two questionnaires and is estimated to take approximately 10 to 15 minutes. The study will take place in the classroom setting, as well as online.

**POTENTIAL RISKS AND DISCOMFORTS:**
We do not anticipate any risks or discomforts to you from participating in this research. If you wish, you may choose to withdraw your consent and discontinue your participation at any time during the study without penalty.

**BENEFITS:**
You will receive no direct benefit from your participation in this study; however, the possible benefits to others include a better understanding of intercultural competence in todays’ society.

**PRIVACY/CONFIDENTIALITY:**
Because you will not be providing any information that can uniquely identify you (such as your name or student ID number), the data you provide will be anonymous.

There is no payment or other form of compensation for your participation in this study.

Your participation is voluntary and you may refuse to participate without penalty or loss of benefits.  Furthermore, you may skip any questions or tasks that make you uncomfortable and may discontinue your participation at any time without penalty or loss of benefits. In addition, the researcher has the right to withdraw you from participation in the study at any time.

Please ask any questions you have now. If you have questions later, you should contact Lioba Gierke at [lioba.gierke@web.de](mailto:lioba.gierke@web.de). If you have questions or concerns about your rights as a participant in this study, you may contact the Dickinson College Institutional Review Board at (717) 245-1309. Additional contact information is available at: <http://www.dickinson.edu/homepage/78/institutional_review_board>

**I HAVE READ THE ABOVE INFORMATION. ANY QUESTIONS I HAVE ASKED HAVE BEEN ANSWERED. I AGREE TO PARTICIPATE IN THIS RESEARCH PROJECT AND I WILL RECEIVE A COPY OF THIS CONSENT FORM.**

*PARTICIPANT'S SIGNATURE DATE*

## Document B. Questionnaire 1

Study “Intercultural Competence”

Spring 2016

Please answer the following questions:

Question 1: Please describe in your own words what “intercultural competence” means to you:

Demographic characteristics:

Question 2: gender: female male

Question 3: age: _____________

Question 4: nationality: ________________________________________

Question 5: Semester you are studying in…

1 2 3 4 5 6 7 8 Other: ____________

Question 6: Major:

Bachelor of Psychology Other: _________________________

Question 7: In which countries have you lived for at least 6 months in your lifetime? _______________________________________________________________________

Question 8: Were you enrolled in an international school? YES NO

Question 9: What was the spoken language at your high school? _______________________

Question 10: Have you ever studied abroad before studying at Dickinson College

YES NO

Question 11: Have you ever participated in an event/workshop/class concerning intercultural topics?

YES NO If yes, what kind of event and for how long? __________

___________________________________________________________________________

Question 12: Have you ever hosted an international exchange student in your home?

YES NO

Thank you!

## Document C. Questionnaire 2

“Intercultural Competence Study”

Spring 2016, Questionnaire 2

Think of an interculturally-competent person. Which characteristics does such an interculturally-competent person possess?

Consider each pair of characteristics. Place a cross on the number that best reflects the extent to which you believe the characteristic describes an interculturally-competent person.

outgoing 1 2 3 4 5 6 shy

inflexible 1 2 3 4 5 6 flexible

extroverted 1 2 3 4 5 6 introverted

reserved 1 2 3 4 5 6 talkative

traditional 1 2 3 4 5 6 progressive

selfish 1 2 3 4 5 6 unselfish

observant 1 2 3 4 5 6 naïve

curious 1 2 3 4 5 6 indifferent

compassionate 1 2 3 4 5 6 discrete

empathetic 1 2 3 4 5 6 unfeeling

including 1 2 3 4 5 6 excluding

disjoint 1 2 3 4 5 6 cooperating

tolerant 1 2 3 4 5 6 intolerant

patient 1 2 3 4 5 6 impatient

open-minded 1 2 3 4 5 6 narrow-minded

respectful 1 2 3 4 5 6 lacking respect

harmonious 1 2 3 4 5 6 disagreeable

unfitting 1 2 3 4 5 6 adaptable

agitated 1 2 3 4 5 6 calm

non-judgmental 1 2 3 4 5 6 judgmental

hostile 1 2 3 4 5 6 amicable

Which of the characteristics above are the THREE most important characteristics of an interculturally-competent person in your opinion? Please write them down in the order of importance to you.

1. ________________________________________________________________

2. ________________________________________________________________

3. __________________________________________________________________

Thank you for your participation!

## Document D. Debriefing form

**“Intercultural Competence Study”**

Thank you for participating in the study on intercultural competence. You were invited to participate because you were identified as an undergraduate student from Dickinson College. The purpose of this study is to determine the understanding of intercultural competence in college students. If you would like to receive a summary of the research findings of this study once it is completed, please feel free to contact Lioba Gierke (lioba.gierke@web.de).

Additionally, as this study is part of a larger investigation, we ask that you please *do not discuss* this study with any other students.

Should you experience any feelings of discomfort subsequent to completing this study, please feel free to contact the Dickinson College Wellness Center at 717-245-1485 to talk to a professional counselor or obtain a psychological evaluation. Additionally, the principal investigator will be available for discussion of this study or related topics immediately following the study.

**Thank you again for your participation!**

# Table A. Dimensions of IC in two samples of undergraduate university students

| **Sample in Germany**  **(Jacobs University Bremen, Germany)^a^ *n* (% of 130)** | **Sample in the USA**  **(Dickinson College, USA)**  ***n* (% of 93)** |
| --- | --- |
| (1) External Outcomes (*n*=102; 78%)   1. Interaction 2. Communication | (1) Knowledge (*n*=47; 51%)   1. Intercultural awareness 2. Understanding other’ world views |
| (2) Attitudes (*n*= 72; 55%)   1. Tolerance/acceptance 2. Respect | (2) External Outcomes (*n*= 26; 28%)   1. Interaction 2. Communication |
| (3) Knowledge (*n*=59; 45%)   1. Intercultural awareness 2. Understanding others’ worldviews | (3) Attitudes (*n*= 22; 24%)   1. Respect 2. Tolerance/acceptance |
| (4) Internal Outcomes (*n*=24; 18%)   1. Adaptability/adjustment 2. Empathy | (4) Internal Outcomes (*n*= 12; 13%)   1. Informed frame of reference 2. Adaptability/adjustment |
| (5) Intrapersonal Skills (*n*=22; 17%)   1. Problem solving 2. Culture detection | (5) Intrapersonal Skills (*n*= 5; 5%)   1. Culture detection 2. Judgment inhibition |
| (6) Interpersonal Skills (*n*=2; 2%)   1. Interactive learning/Observation | (6) Interpersonal Skills (*n*= 2; 2%)   1. Interpersonal skills miscellaneous |

Note. Dimensions of IC are arranged by importance in each sample. The most frequently mentioned subcategories are listed for each dimension.

^a^The sample in Germany was described in the following study: Odag O, Wallin HR, Kedzior KK. Definition of intercultural competence according to undergraduate students at an international university in Germany. J Stud Int Educ. 2016; 20(2):118-139.

# Table B. Comparison in dimensions of IC between two samples of undergraduate university students

| **Characteristics** | **Sample in Germany^a^**  ***n* (% of 130)** | **Sample in the USA**  ***n* (% of 93)** | **Test** | | |
| --- | --- | --- | --- | --- | --- |
|  |  |  | ***χ^2^* (*df*)^b^** | ***p*-value** | **Cramer’s *V^c^*** |
| **Demographics** |  |  |  |  |  |
| Nationality  Domestic  International  Study abroad experience before attending university  No  Yes  Took part in intercultural workshops/courses  No  Yes | 55 (42%)  75 (58%)  75 (58%)  55 (42%)  0 (0%)  130 (100%) | 71 (76%)  22 (24%)  72 (77%)  21 (23%)  36 (39%)  57 (61%) | 24.19 (1)  8.53 (1) | **<.001***  **.003*** | .34  .21 |
| **IC dimensions** |  |  |  |  |  |
| Attitudes  No  Yes  External Outcomes  No  Yes  Knowledge  No  Yes  Internal Outcomes  No  Yes  Intrapersonal Skills  No  Yes  Interpersonal Skills  No  Yes | 58 (45%)  72 (55%)  28 (22%)  102 (78%)  71 (55%)  59 (45%)  106 (82%)  24 (18%)  108 (83%)  22 (17%)  128 (98%)  2 (2%) | 71 (76%)  22 (24%)  67 (72%)  26 (28%)  46 (49%)  47 (51%)  81 (87%)  12 (13%)  88 (95%)  5 (5%)  91 (98%)  2 (2%) | 21.10 (1)  54.51 (1)  .39 (1)  .86 (1)  5.75 (1)  <.001 (1) | **<.001***  **<.001***  .533  .354  **.016***  1.000 | .32  .50  .05  .07  .18  .02 |

Note. ^a^The sample in Germany was described in the following study: Odag O, Wallin HR, Kedzior KK. Definition of intercultural competence according to undergraduate students at an international university in Germany. J Stud Int Educ. 2016; 20(2):118-139. ^b^Pearson’s chi-square with continuity correction for 2×2 comparisons. Cramer’s *V* is a measure of an effect size on a scale from 0 to 1 and the same interpretation as that used for Pearson correlation coefficients (< .3 is a small effect, < .5 medium effect, > .5 large effect).

# Table C. Ranks of the most important characteristics of an interculturally-competent person

| Characteristic | Rank 1 | Rank 2 | Rank 3 | Overall Rank | % of *n* = 93 |
| --- | --- | --- | --- | --- | --- |
| Open-minded | 23 | 16 | 21 | 60 | 64% |
| Respectful | 13 | 8 | 11 | 32 | 34% |
| Observant | 7 | 7 | 11 | 25 | 27% |
| Tolerant | 9 | 10 | 5 | 24 | 26% |
| Curious | 7 | 9 | 5 | 21 | 23% |

# Figure A. Heatmap of a four-cluster solution


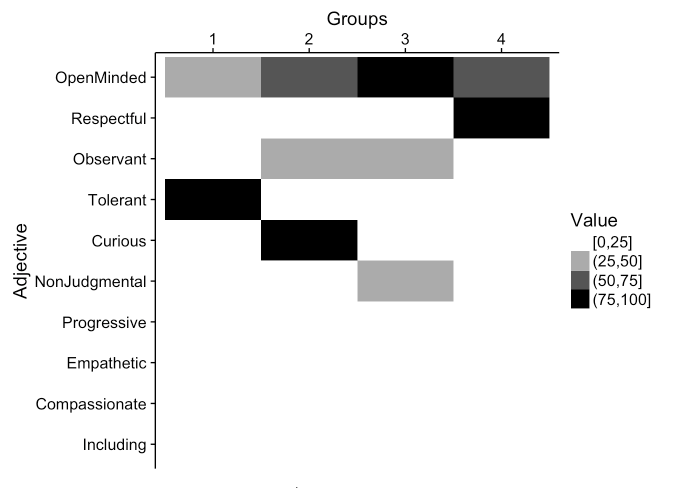


Note. ‘Value’ corresponds to the loading of each adjective onto each group (cluster).
