# Supplementary material for: Definition of intercultural competence (IC) in undergraduate students at a private university in the USA: A mixed-methods study
Source: PLoS One. 2018 Apr 26;13(4):e0196531. doi: 10.1371/journal.pone.0196531 (PMC5919648; doi:10.1371/journal.pone.0196531)
Supplement: S2 File — (ZIP) [file pone.0196531.s002.zip › IC_USA_Data Coding Manual.pdf]

Coding Manual

Variable	Meaning
Q1	Instructions: Definition of intercultural competence
Gender	1=male; 2=female
Age	Age
US_nationality	1=yes; 2=no
Countries	Have you lived in other countries than the USA for at least 6 months?<br>1=yes (other country only or USA and other country), 2=no (only USA)
International_school	1=yes; 2=no
English_school	Spoken language at high school 1=English; 2=other (or English+other)
StudyAbroad	1=yes; 2=no
ICTopics	1=yes; 2=no
Adjective pairs:	
P1	Outgoing – Shy
P2	Inflexible – Flexible
P3	Extroverted – Introverted
P4	Reserved – Talkative
P5	Traditional – Progressive
P6	Selfish – Unselfish
P7	Observant – Naïve
P8	Curious – Indifferent
P9	Compassionate – Discrete
P10	Empathetic – Unfeeling
P11	Including – Excluding
P12	Disjoint – Cooperating
P13	Tolerant – Intolerant
P14	Patient – Impatient
P15	Open-minded – Narrow-Minded
P16	Respectful – Lacking Respect
P17	Harmonious – Disagreeable
P18	Unfitting – Adaptable
P19	Agitated – Calm
P20	Non-judgmental – Judgmental
P21	Hostile - Amicable
Characteristics	Description of task: three most important characteristics of an interculturally-competent person selected from all given adjectives<br><br>Note that #NULL! refers to empty cells (ie. the characteristic not selected in the list of top 3 adjectives)
